# High Viral Load and Poor Ventilation: Cause of High Mortality From COVID-19

**DOI:** 10.1177/1010539520944725

**Published:** 2020-07-25

**Authors:** Shyam Aggarwal, Shreyas Aggarwal, Anita Aggarwal, Kriti Jain, Sachin Minhas

**Affiliations:** 1 Sir Ganga Ram Hospital, New Delhi, India; 2 Kasturba Medical College, Mangalore, Karnataka, India; 3 Max Smart Super Speciality Hospital, New Delhi, India

The COVID-19 pandemic has a high mortality that varies between countries. The differences in mortality rates seem to be higher in higher income countries compared with lower and middle-income countries (World Health Organization reports). There may be a number of reasons for this including case definitions used and reporting and testing rates. The reason for the difference is not understood. In this article, we have tried to explain that poor ventilation and subsequent buildup of high viral load could be a reason for such a drastic difference in mortality rates among these two groups of countries.

Liu et al^1^ published their results in *Lancet Infect Diseases*, March 2020, that the mean viral load of severe cases was around 60 times higher than that of mild cases. They calculated the viral load by Δct method. Chung-Ming Chu et al published in *CMAJ* in 2004 their data of 32 patients demonstrating the evidence that higher initial viral load in the nasopharyngeal specimen was independently associated with worse prognosis in SARS.^2^ These studies suggest an association of high viral load in nasal cavity and nasopharynx with the severity of the disease.

## Role of Ventilation in Controlling Airborne Transmission

Airborne transmission plays a crucial role in the spread of the virus. This route of transmission has also been recently acknowledged by international agencies including the World Health Organization, China National Health Commission, and Japanese authorities. Ventilation is considered to be an important determinant in the transmission of airborne infections. This fact was reiterated by the SARS outbreak in 2003.^3^ In a review article by Morawska and Cao,^4^ it was emphasized that the airborne transmission route of SARS-CoV-2 has been underplayed so far. The authors stressed on the role of increasing ventilation rate using natural ventilation, avoiding air recirculation, avoiding staying in another person’s direct air flow, and reducing the number of people sharing the same room. The absence of efficient fresh air ventilation in the homes, offices, and hospitals leads to enhanced risk of transmission of viruses including SARS, SARS-CoV-2. This appears to be the key factor determining the cumulative viral load in the nasal cavity and nasopharynx of the people living or working in poorly ventilated closed spaces.

Our review of literature suggests that many people living in higher income countries spend more time indoors (offices and homes) often in centrally heated or air-conditioned premises where they could be exposed to a higher viral load. This is especially true if there are asymptomatic COVID-19-infected individuals in the immediate environment. Under these conditions, the virus may persist in respiratory droplets or in the environment for a long time. This may further lead to a continuous and regular exposure on daily basis that could result in a higher viral load in the nasal cavity and nasopharynx. This high viral load is responsible for the development of severe respiratory disease including acute respiratory distress syndrome and increase the mortality risk. By contrast, people in low-income countries (south and southeast Asian countries) are not exposed to such viral concentrations due to their traditional lifestyle of residing in homes and working in offices with open air ventilation (partly due to lack of resources). For this reason, the viral load in the nasal cavity and the nasopharynx could be lower and result in a less severe disease. We believe that this is a hypothesis worth exploring further.
